# Enhancing nursing competency through virtual reality simulation among nursing students: a systematic review and meta-analysis

**DOI:** 10.3389/fmed.2024.1351300

**Published:** 2024-05-07

**Authors:** Mi-Kyoung Cho, Mi Young Kim

**Affiliations:** ^1^Department of Nursing Science, Chungbuk National University, Cheongju, Republic of Korea; ^2^College of Nursing, Hanyang University, Seoul, Republic of Korea

**Keywords:** virtual reality, simulation, nursing students, competency, meta-analysis

## Abstract

**Aim:**

Studies on the effectiveness of virtual reality (VR) in nursing education have explored its impact on learning outcomes, emotional immersion and engagement, learner self-confidence, and satisfaction, generally showing positive aspects. However, there is a need for a systematic review to examine the specific influence of VR-based education on nursing students’ practical competency.

**Method:**

According to the PRISMA 2020 guidelines, 22 studies were selected based on inclusion criteria from 579 articles, published from January 1, 2018, to March 31, 2024, across nine major databases including PubMed and EMbase. The target population comprised nursing students, and the intervention focused on VR-based simulations aimed at enhancing competency, compared to control groups receiving either no intervention or conventional non-virtual simulation. The primary outcome, nursing competency, was analyzed using MIX 2.0 Pro (Ver. 2.0.1.6, BiostatXL, 2017) to calculate pooled effect sizes.

**Result:**

The pooled effect size for nursing competency was determined to be large, with Hedge’s *g* = 0.88 (95% CI, 0.47 to 1.29). Meta-regression analysis identified several factors associated with an increase in nursing competency. These included studies published after 2022, approval of an IRB, absence of funding, randomized controlled trials (RCTs), interventions reported as shorter than 4 weeks or not reported, sessions fewer than 4 or not reported, session duration under 1 h or not reported, and observational measurement methods. Additional factors enhancing nursing competency were the inclusion of a pre-briefing before simulations, the absence of a debriefing afterward, and the exclusion of other activities during the simulation.

**Conclusion:**

By combining the results of the included studies, the systematic review and meta-analysis accounted for variations in sample size, study methodology, and independent intervention effects, providing an overall evaluation of the effectiveness of simulation-based education in improving nursing students’ competency.

**Limitation:**

The selection criteria for the studies analyzed, which included only those published in English or Korean and reported precise means, standard deviations, and sample sizes, could lead to selection bias and limit the generalization of our study results.

**Systematic review registration:**

PROSPERO International Prospective Register of Systematic Reviews: http://www.crd.york.ac.uk/PROSPERO/, identifier CRD42023446348.

## 1 Introduction

Nursing education is an applied discipline in which theory and practical education are combined; prospective nurses prepare to become competent by applying the knowledge learned in theoretical education to the practical education process. The need for nursing education to train professionals who provide nursing and medical services to humans utilizing digital-based, non-face-to-face media such as artificial intelligence (AI) and big data has recently become more urgent (1). In nursing education, there has been an increasing interest in virtual-reality simulation (VRS) education as an alternative and complementary method to traditional simulation education, providing students with new learning experiences in a reproduced clinical environment and enhancing clinical adaptability (2). Virtual reality (VR) is defined as “the use of partial immersion through a digital learning environment (computer, tablet, phone, screen, etc.) to foster a perceived lived experience for an intended outcome (e.g., learning and entertainment)” (3). This study defines VRS to include VR and its derivatives, augmented reality (AR), and mixed reality (MR), using the terminology consistently. In VRS, learners can collaborate with other healthcare professionals to provide interventions, such as solving patients’ problems or practicing simple skills (4, 5). Improved clinical performance skills, knowledge, and metacognition, as well as enhanced learning satisfaction, communication, self-efficacy, confidence, and teamwork have been reported as effects of these VR programs (4, 6). In addition, studies on the effectiveness of nursing education using VR have been conducted on learning effectiveness, emotional engagement and immersion, learner confidence, and satisfaction (7, 8). Reportedly, VRS programs for nursing skills are effective in improving skills (9) and have the advantage of enabling safe and repetitive training without time and space constraints (10). Thus, learning through VRS has demonstrated improvement in various factors related to clinical nursing competency, albeit often assessed in a fragmented manner. As various forms of VRS are being applied in nursing education, and diverse elements contributing to nursing competency are considered, there is a need to comprehend the holistic outcomes of these studies. Consequently, this study aims to comprehensively review the results, considering nursing competency in a broader sense that encompasses collaboration, interpersonal relationships, communication, professional development, and the nursing process, skills, and education (11).

Moreover, a systematic review and analysis of nursing students’ outcomes are essential for determining specific factors that are deemed effective. Systematic reviews and meta-analyses can amalgamate the results of included studies, accounting for differences in sample size, variations in research approaches, and intervention effects among independent studies. We believe that the systematic review and meta-analysis in this study will enable an assessment of the overall effect of VRS-based education on nursing students’ nursing competency. Consequently, this study aims to provide foundational data on VRS by conducting a systematic literature review and meta-analysis, investigating the improvement effect of VRS on nursing students’ nursing competency as a primary outcome, and examining knowledge, self-efficacy, problem-solving skills, confidence, and satisfaction as secondary outcomes.

This study aims to acquire and analyze evidence regarding the enhancement of nursing students’ nursing competency through VRS. The primary outcome focuses on nursing students’ self-reported feelings and reactions, while the secondary outcome assesses nursing students’ nursing competency following exposure to VRS.

## 2 Materials and methods

### 2.1 Search strategy and data sources

The search was jointly conducted by two researchers, Cho, M.-K. and Kim, M.Y., across nine electronic databases or e-journals: PubMed, Cochrane, EMBASE-OVID, CINAHL, World of Science, SCOPUS, PQDT, APA PsycArticles, and Research Information Sharing Service. The primary search, conducted from July 18, 2023, to August 20, 2023, targeted articles published in English and Korean from January 1, 2003, to April 30, 2023. A secondary search was carried out from April 6, 2024, to April 9, 2024, focusing on articles published from May 1, 2023, to March 31, 2024, also in English and Korean. The search strategy and formula, following the PICO-SD framework (population, intervention, comparison, outcome, study design), are detailed in Table 1. The keywords employed in search terms across the nine databases included combinations and variations of “nursing students,” “virtual reality,” “augmented reality,” “extended reality,” “metaverse,” “competency-based education,” “clinical competence,” “competency,” and “controlled clinical trial.” These keywords were chosen to comprehensively capture studies relevant to the impact of virtual reality simulation on nursing competency.

**Table 1:** Search strategy according to PICO.

PICO	Key terms	MeSH	PubMed Entry Terms	EMTREE (EMBASE)	Text words
P (Patient, Population, Participants, Problems)	Nursing student(s)	“Students, Nursing”[Mesh]	Pupil Nurses Student, Nursing Nurses, Pupil Nurse, Pupil Pupil Nurse Nursing Student Nursing Students	Nursing student/	[(student* OR pupil*) AND nurs*]
I (Intervention or Exposure or Index Test)	Virtual reality	“Virtual Reality”[Mesh]	Reality, Virtual Virtual Reality, Educational Educational Virtual Realities Educational Virtual Reality Reality, Educational Virtual Virtual Realities, Educational Virtual Reality, Instructional Instructional Virtual Realities Instructional Virtual Reality Realities, Instructional Virtual Reality, Instructional Virtual Virtual Realities, Instructional	Virtual reality/	[(educational OR instructional) AND virtual realit*]
	Augmented reality	“Augmented Reality”[Mesh]	Augmented Realities Realities, Augmented Reality, Augmented Mixed Reality Mixed Realities Realities, Mixed Reality, Mixed	Augmented reality/	(augmented OR mixed) AND realit*
	Mixed reality				
	Extended reality	–		–	Extended realit*
	Metaverse	–		–	Metaverse OR meta-verse
C (Comparators, Comparisons, Controls)	None or usual				
O (Outcomes, Effects)	Competency	“Competency-Based Education” [Mesh]	Competency-based education education, competency-based competency-based educations education, competency-based educations, competency-based	–	
		“Clinical Competence”[Mesh]	competency, clinical competence, clinical clinical competency clinical competencies competencies, clinical clinical skill skill, clinical skills, clinical clinical skills	clinical competence/	Clinical compete*
Study Design	RCT, Quasi-experimental	“Controlled Clinical Trials as Topic”[Mesh]	Clinical Trials, Controlled as Topic	Controlled clinical trial (topic)/OR Controlled Clinical Trials as Topic.mp.	
Restrictions	English, Korean/Humans (Adult: 19+ years), (Young Adult 19–24 years) Male, Female/1900.01.01–2024.03.31				

### 2.2 Inclusion and exclusion criteria

The reporting of the results adhered to the PRISMA 2020 checklist. Inclusion criteria comprised nursing students aged 19 years or older (Population), interventions involving VRS (Intervention), with conventional learning methods or no intervention as the control (comparison). The primary outcome was nursing competency, and secondary outcomes included knowledge, self-efficacy, problem-solving, confidence, satisfaction, and other variables, which were concurrently measured. If multiple measurements were conducted post-intervention, the first measurement was used to calculate the effect size. Only studies presenting subject numbers, means, and standard deviations in the results were selected for precise effect-size calculation. The study designs included randomized controlled trials (RCTs) and quasi-experimental studies. Exclusion criteria included studies encompassing students from majors other than nursing, interventions using conventional simulation-learning methods instead of VRS, the absence of nursing competency as an outcome variable, studies not reported in Korean or English, studies with inaccessible original texts, and single-group studies lacking a control group.

### 2.3 Data extraction

Two researchers, Cho, M.-K. and Kim, M.Y., independently conducted searches and selected studies for analysis based on the predefined inclusion and exclusion criteria. The selected studies were extracted, incorporating information such as author, year of publication, country, publication language, number of schools, institutional review board (IRB) approval, funding details, number of participants, study design, intervention characteristics (type, facilitator, duration, session, time/session, pre-briefing, debriefing, other activities, outcome measurement time, and measurement method), quality assessment score, and dependent variables. This information was meticulously recorded in a coding book created using the Microsoft Excel spreadsheet software. Any disparities in coding were addressed by revisiting the original text to ascertain and input the final coding values (Table 2).

**Table 2:** Descriptive summary of the included studies.

Study ID	Author (Year)	Country	Center	IRB	Fund	Research design	Participants	Intervention type	Program facilitator	Intervention duration	Intervention session	Intervention time/session	Outcome measurement time	outcome variable	Pre-briefing	Debriefing	Non-simulation activities	Quality score
1	Lee (12)	Korea	1	Yes	Yes	Quasi	40 Senior nursing students from a nursing college (E: 20, C:20)	Virtual reality simulation (VRS)	Researcher	None reported	None reported	80 min	Delayed (3 days after interventions for each team)	-Knowledge -Performance confidence -Clinical practice competency	Yes	Yes	None	8
2	Ahn and Lee (13)	Korea	2	Yes	No	Quasi	84 Nursing students (E: 44, C: 40)	Virtual reality simulation (VRS)	Nursing faculty	1 day	1 session	35–50 min	Immediately	-Knowledge -Confidence -Self-efficacy -Clinical competency	Yes	Yes	None	8
3	Rossler et al. (14)	USA	1	Yes	Yes	RCT	20 Prelicensure baccalaureate nursing students (E: 5, C: 15)	Virtual reality simulation (VRS)	Investigator	None reported	None reported	None reported	Delayed (1 week)	-Knowledge of OR fire safety -Transfer of knowledge of OR fire safety skills	Yes	None	None	4
4	Aebersold et al. (15)	USA	1	Yes	Yes	RCT	69 Sophomore and junior nursing students (E: 35, C: 34)	Virtual reality simulation (VRS)	None reported	Over 4 weeks	None reported	None reported	Immediately	-Skill competency evaluation	Yes	Yes	None	8
5	An et al. (16)	Korea	2	Yes	No	RCT	62 First- and second-year nursing students (E: 31, C:31)	Virtual reality simulation (VRS)	Researcher	4 weeks	None reported	None reported	Immediately	- Self-regulated learning competency - Perceived learning competency - Knowledge - Learning flow - Academic stress	Yes	None	None	11
6	Chang et al. (17)	Taiwan	1	No	Yes	Quasi	42 Two classes at a nursing university (E: 21, C:21)	Virtual reality simulation (VRS)	Nursing faculty	3 weeks	None reported	None reported	Immediately	- OSCE competency - Problem-solving skills - Learning engagement - Learning satisfaction	None	None	None	6
7	Ahn (11)	Korea	1	Yes	No	Quasi	72 second-year nursing students (E: 34, C:38)	Metaverse based simulation	Nursing faculty	None reported	1 session each	25–35 min	Immediately	- Knowledge of core nursing skills - Confidence in core nursing skill performance -Clinical competency	Yes	Yes	None	8
8	Kim and Jung (18)	Korea	1	Yes	No	RCT	73 First- and second-year nursing students (E: 37, C:36)	Virtual reality simulation (VRS)	Researcher	None reported	None reported	30 min	Immediately	-Clinical competency -Self-efficacy -Satisfaction	Yes	None	None	9
9	Ha et al. (19)	Korea	1	No	No	RCT	70 Third-year nursing students (E: 35, C: 35)	Virtual reality simulation (VRS)	Researcher	None reported	None reported	2 h	Immediately	-Clinical competency -Self-efficacy -Nursing skill competency -Satisfaction	None	None	None	10
10	Yoo and Yang (20)	Korea	1	Yes	No	Quasi	48 Second-year nursing students (E: 24, C: 24)	Virtual reality simulation (VRS)	Researcher	5 weeks	5 sessions	20–30 min/once	Immediately	- Clinical competency - Problem-solving skills - Confidence in core nursing skill performance	Yes	Yes	None	7
11	Bae and Shin (21)	Korea	1	Yes	No	Quasi	45 Fourth-year nursing student (E: 24, C: 21)	Virtual reality simulation (VRS)	Researcher	None reported	None reported	35 min	Immediately	- Clinical performance competency - Problem-solving skill - Confidence in performance	Yes	Yes	None	8
12	Song (22)	Korea	1	No	No	Quasi	117 Third-year nursing student (E: 58, C: 59)	Virtual reality simulation (VRS)	Nursing faculty	10 days	10 sessions	8 h/day	Immediately	-Competencies of socio-emotion - Psychiatric nursing competency - Learning self-efficacy - Transition synchronization -Social distance	None	None	Yes	7
13	Raman et al. (23)	Oman	1	Yes	Yes	Quasi	74 Fourth-year nursing student (E: 34, C: 40)	Virtual reality simulation (VRS)	Nursing faculty	34 h of HFS + 101 h of TCT	None reported	None reported	Immediately	-Clinical competencies -Knowledge levels among nursing students	Yes	Yes	None	8
14	Cho et al. (24)	Korea	1	Yes	Yes	Quasi	69 Senior nursing students (E: 36, C: 33)	Metaverse-based simulation	Researcher	1 day	None reported	1 h	Immediately	-Competency -Self-efficacy -Learning realism -Learning satisfaction	Yes	Yes	Yes	9
15	Lee and Baek (25)	Korea	1	Yes	No	Quasi	44 Third-year nursing students (E: 22, C: 22)	Virtual reality simulation (VRS)	Researcher	2 weeks	None reported	2 h of VRS + 4 h of HFS	Immediately	-Performance confidence -Clinical decision-making ability	Yes	Yes	None	9
16	Kim and Heo (26)	Korea	2	Yes	Yes	Quasi	63 Sophomore nursing students (E: 33, C: 30)	Augmented reality	Researcher	2 weeks	2 sessions	2 h	Immediately	-Learning satisfaction -Skill competency -Confidence in medication safety	Yes	None	None	8
17	Park and Yoon (27)	Korea	1	Yes	No	Quasi	44 Second-year students (E: 22, C: 22)	Virtual reality simulation (VRS)	Researcher	3 weeks	3 sessions	30 min	Immediately	-Nursing skills -Performance confidence -Learning satisfaction	Yes	None	None	9
18	Sahin Karaduman and Basak (28)	Turkey	1	Yes	No	RCT	126 Third-year nursing students (E1: 42, E2: 42, C: 42)	Virtual patient simulations	Researcher	None reported	2 sessions	15 min	Immediately	-Nursing anxiety -Self-confidence -Learning evaluation -Performance	Yes	Yes	None	10
19	Moon (29)	Korea	1	Yes	Yes	Quasi	72 Third-year nursing students (E: 34, C: 38)	Metaverse based program	Nursing faculty	1 day	1 session	3 h	Immediately	-Clinical competency -Problem solving efficacy -Learning satisfaction	Yes	No	Yes	8
20	Lee (30)	Korea	1	Yes	No	Quasi	48 Senior nursing students (E: 24, C: 24)	Virtual reality simulation (VRS)	Nursing faculty	None reported	None reported	3 h	Immediately	-Critical thinking disposition -Clinical competency -Self-efficacy	Yes	Yes	No	8
21	Ahn (31)	Korea	1	Yes	No	Quasi	70 Nursing students (E: 34, C: 36)	Metaverse based training	Nursing faculty	1 day	1 session	10–15 min	Immediately	-Performance confidence -Performance ability	Yes	Yes	No	7

IRB, Institutional Review Board; USA, United States of America; E, experimental group; C, control group; RCT, randomized controlled trial; Quasi, quasi-experimental study; HFS, high fidelity stimulation. TCT, traditional clinical training; OSCE, objective structured clinical examination; OR, operating room.

### 2.4 Quality assessment

The quality assessment of selected articles was independently performed by Cho, M.-K. and Kim, M.Y. using the Joanna Briggs Institute (JBI) Checklist for RCTs and the Checklist for Quasi-Experimental Studies. Five RCTs were assessed using the 13-question JBI Checklist; the average score was 8.40, and all five studies lacked clear reporting on “Q2. Was allocation to treatment groups concealed?” and “Q4. Were participants blind to treatment assignment?” Quasi-experimental studies comprised eight articles, and on evaluation using the 9-item JBI Checklist for Quasi-Experimental Studies (32), the average score was 8.50, with generally well-reported items (Table 3).

**Table 3:** Quality assessment of the included studies.

Study ID	Joanna Briggs Institute of Critical Appraisal Tools Checklist for checklist for randomized controlled trials													Total score
	Q1	Q2	Q3	Q4	Q5	Q6	Q7	Q8	Q9	Q10	Q11	Q12	Q13	
3	0	0	0	0	0	1	0	1	0	1	1	0	0	4
4	0	0	1	0	1	1	1	1	1	1	1	0	0	8
5	1	0	1	0	1	1	1	1	1	1	1	1	1	11
8	1	0	1	0	0	1	0	1	1	1	1	1	1	9
9	1	0	1	0	0	1	1	1	1	1	1	1	1	10
18	1	0	1	0	1	0	1	1	1	1	1	1	1	10
Total	4	0	5	0	3	5	4	6	5	6	6	4	4	8.67

Study ID	Joanna Briggs Institute of Critical Appraisal Tools Checklist for quasi-experimental study									Total score
	Q1	Q2	Q3	Q4	Q5	Q6	Q7	Q8	Q9	
1	1	1	1	1	1	1	1	1	1	9
2	1	1	1	1	1	1	1	1	1	9
6	1	1	1	1	1	0	1	1	0	7
7	1	1	1	1	1	1	1	1	1	9
10	1	1	1	1	1	0	1	1	1	8
11	1	1	1	1	1	1	1	1	1	9
12	1	1	1	0	1	1	1	1	1	8
13	1	1	1	1	1	1	1	1	1	9
14	1	1	1	1	1	1	1	1	1	9
15	1	1	1	1	1	1	1	1	1	9
16	1	1	1	1	1	0	1	1	1	8
17	1	1	1	1	1	1	1	1	1	9
19	1	1	1	1	0	1	1	1	1	8
20	1	1	1	1	0	1	1	1	1	8
21	1	0	1	1	0	1	1	1	1	7
Total	15	14	15	14	12	12	15	15	14	8.40

### 2.5 Statistical analyses

MIX 2.0 Pro (Ver. 2.0.1.6, BiostatXL, 2017) was used to calculate and merge effect sizes for both the primary outcome of nursing competency and secondary outcomes. The overall effect was determined using a random-effects model, considering between-subject variability and heterogeneity between studies. Hedge’s g was employed for effect-size calculation, and significance was assessed using 95% confidence intervals (CIs), *Z* tests, and *p-*values. The weight of each effect size was determined using the inverse of variance (33). Heterogeneity was evaluated using Higgin’s *I*^2^ (34), with an *I*^2^ of >50% indicating heterogeneity (35). Subgroup analysis, meta-regression, and exclusion-sensitivity analysis were conducted for nursing competency to identify factors contributing to heterogeneity. Publication bias was examined using funnel plots, trim-and-fill plots, Begg’s test, Egger’s regression, and the trim-and-fill method to correct for the overall effect (36).

## 3 Results

### 3.1 Characteristics of the included studies

**Figure 1:**
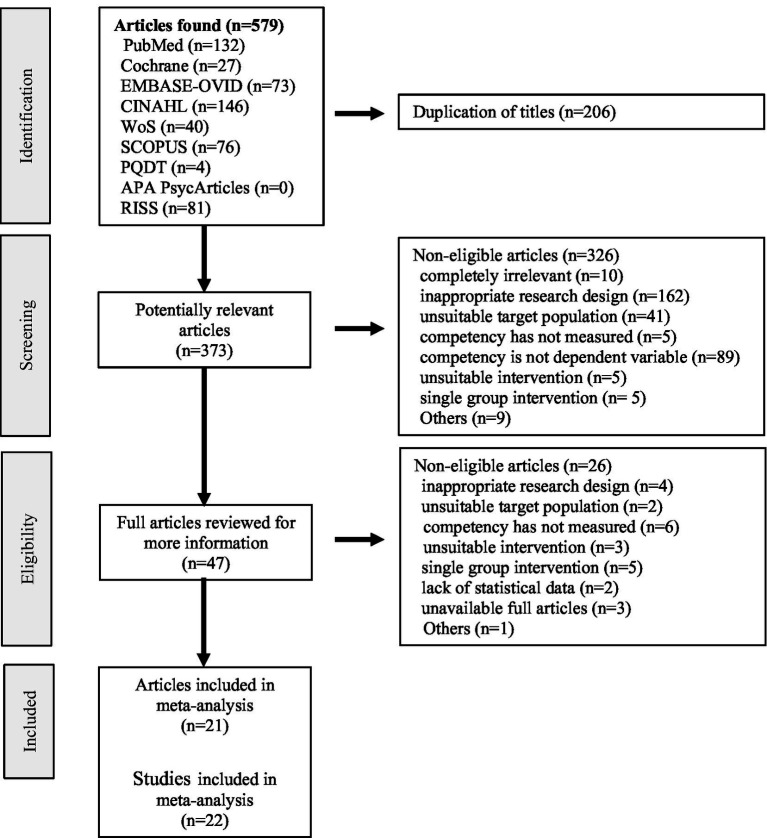
PRISMA flow diagram. An article by Sahin Karaduman and Basak (28), designed using two experimental groups, was divided into two studies.

A total of 579 articles were initially identified from 9 databases following the search strategy. After excluding duplicates, 373 articles were extracted. Following the application of the inclusion and exclusion criteria, 21 research articles were ultimately selected. The research by Sahin Karaduman and Basak (28) was designed using two experimental groups and was analyzed as two separate studies, resulting in 22 studies being analyzed (Figure 1). Of these, six studies were published before 2022; three were conducted in the United States (USA), twelve studies were published in English; nineteen were conducted at a single university; nineteen and nine studies had IRB approval and funding, respectively. The study designs included seven RCTs, fifteen quasi-experimental studies, and eight studies with fewer than 60 participants. Interventions comprised 18 VR/AR simulations and four metaverse. Eight studies had a professor as a facilitator, four had an intervention duration of more than 4 weeks, two had four or more intervention sessions, eight had an intervention time of more than 1 h per session, 19 had a pre-briefing, and nine had a debriefing. Dependent-variable measurements were taken immediately after the intervention in 20 studies, the measurement method was observational measurement in 12 studies, 19 studies had no additional activities, such as reflection, besides the simulation, and 14 studies had an above-average quality assessment score (Table 2).

### 3.2 Effect of VRS-based intervention on nursing competency

**Figure 2:**
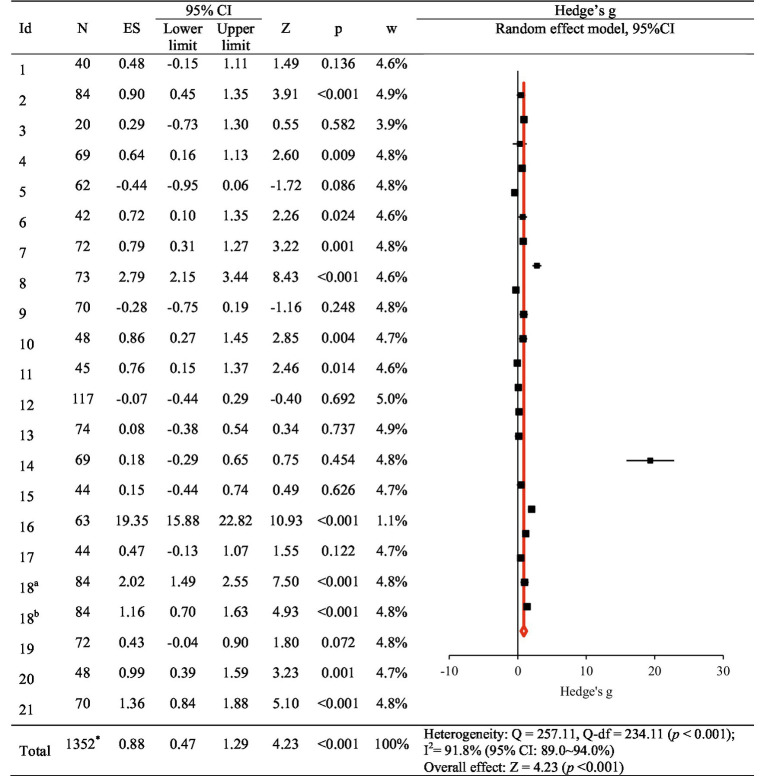
The effect of virtual reality simulation-based intervention on nursing competency. ES, effect size; CI, confidence interval. An article by Sahin Karaduman and Basak (29), designed using two experimental groups, was divided into two studies (18^a^ and 18^b^). *Removal of the number of duplicate subjects in the 18th study.

The overall effect of nursing competency, as the primary outcome for the 22 VRSs, was found to be Hedge’s *g* = 0.88 (95% CI: 0.47 to 1.29). This was interpreted as a large effect based on the criteria provided by Brydges (37) for interpreting effect sizes (Figure 2). The high degree of heterogeneity among the studies, indicated by Higgins’s *I*^2^ of 91.8% in the heterogeneity test, prompted subgroup and meta-regression analyses to explore factors contributing to this heterogeneity.

In subgroup analyses, the characteristics of studies significantly associated with improvements in nursing competency IRB-approved studies (Hedge’s *g* = 1.02, 95% CI: 0.57, 1.48); interventions with a duration not reported or those with a duration of less than 4 weeks (Hedge’s *g* = 1.05, 95% CI: 0.56, 1.53); interventions with sessions not reported or those with less than 4 sessions (Hedge’s *g* = 0.95, 95% CI: 0.50, 1.39); those with outcome measurement immediately after the intervention (Hedge’s *g* = 0.93, 95% CI: 0.50, 1.37); those with pre-briefing before the simulation (Hedge’s *g* = 0.71, 95% CI: 0.23, 1.20); those with debriefing after the simulation (Hedge’s *g* = 1.02, 95% CI: 0.57, 1.48); and those with no other activities besides the simulation, such as keeping a reflective journal (Hedge’s *g* = 1.03, 95% CI: 0.55, 1.50). Publication year, Country, publication language, number of schools, funding status, research design, number of participants, intervention type, facilitator, intervention time per session, measurement method, debriefing, and quality assessment score also showed statistically significant effect sizes (Table 4).

**Table 4:** Subgroup analysis of nursing competency according to study characteristics.

Variables	Category	*K*	Study ID	*N*	ES	95% CI		*Z*	*p*-value
						Lower limit	Upper limit		
Year	2018 ~ 2021	6	2, 3, 4, 10, 12, 13	412	0.44	0.06	0.82	2.29	0.022
	≥2022	16	1, 5, 6, 7, 8, 9, 11, 14, 15, 16, 17, 18^a^, 18^b^, 19, 20, 21	940	1.12	0.56	1.68	3.93	<0.001
Country	Beyond the USA	19	1, 2, 5, 6, 7, 8, 9, 10, 11, 12, 13, 14, 15, 16, 18^a^,18^b^, 19, 20, 21	1,219	0.96	0.50	1.43	4.08	<0.001
	USA	3	3, 4, 17	133	0.54	0.19	0.89	3.00	0.003
Language	Korean	10	2, 7, 8, 9, 10, 11, 12, 16, 19, 21	714	1.49	0.70	2.28	3.68	<0.001
	English	12	1, 3, 4, 5, 6, 13, 14, 15, 17, 18^a^, 18^b^, 20	638	0.57	0.18	0.95	2.91	0.004
School	1	19	1, 3, 4, 6, 7, 8, 9, 10, 11, 12, 13, 14, 15, 17, 18^a^, 18^b^, 19, 20, 21	1,143	0.72	0.40	1.05	4.36	<0.001
	2	3	2, 5, 16	209	5.23	1.98	8.48	3.15	0.002
IRB	No	3	6, 9, 12	229	0.08	−0.42	0.58	0.30	0.764
	Yes	19	1, 2, 3, 4, 5, 7, 8, 10, 11, 13, 14, 15, 16, 17, 18^a^, 18^b^, 19, 20, 21	1,123	1.02	0.57	1.48	4.39	<0.001
Fund	No	13	2, 5, 7, 8, 9, 10, 11, 12, 17, 18^a^, 18^b^, 20, 21	859	0.86	0.39	1.32	3.61	<0.001
	Yes	9	1, 3, 4, 6, 13, 14, 15, 16, 19	493	1.07	0.28	1.87	2.66	0.008
Research design	Quasi-E	15	1, 2, 6, 7, 10, 11, 12, 13, 14, 15, 16, 17, 19, 20, 21	932	0.85	0.38	1.31	3.59	<0.001
	RCT	7	3, 4, 5, 8, 9, 18^a^,18^b^	420	0.89	0.03	1.75	2.02	0.044
Participants	< 60	8	1, 3, 6, 10, 11, 15, 17, 20	331	0.62	0.39	0.84	5.40	<0.001
	≥ 60	14	2, 4, 5, 7, 8, 9, 12, 13, 14, 16, 18^a^, 18^b^, 19, 21	1,021	1.13	0.53	1.72	3.71	<0.001
Intervention type	Metaverse	4	7, 14 19, 21	283	0.68	0.20	1.17	2.75	0.006
	AR/VR	18	1, 2, 3, 4, 5, 6, 8, 9, 10, 11, 12, 13, 15, 16, 17, 18^a^, 18^b^, 20	1,069	0.97	0.47	1.47	3.78	<0.001
Facilitator	Researcher	14	1, 3, 4, 5, 8, 9, 10, 11, 14, 15, 16, 17, 18^a^, 18^b^	773	1.16	0.50	1.82	3.46	0.001
	Nursing faculty	8	2, 6, 7, 12, 13, 19, 20, 21	579	0.63	0.27	0.98	3.45	0.001
Intervention duration	Not reported or < 4 weeks	18	1, 2, 3, 6, 7, 8, 9, 11, 12, 14, 15, 16, 17, 18^a^,18^b^, 19, 20, 21	1,099	1.05	0.56	1.53	4.24	<0.001
	≥ 4 weeks	4	4, 5, 10, 13	253	0.27	−0.28	0.83	0.97	0.333
Intervention session	Not reported or < 4sessions	20	1, 2, 3, 4, 5, 6, 7, 8, 9, 11, 13, 14, 15, 16, 17, 18^a^,18^b^, 19, 20, 21	1,187	0.95	0.50	1.39	4.17	<0.001
	≥ 4sessions	2	10, 12	165	0.36	−0.55	1.28	0.78	0.436
Intervention time/session	Not reported or < 1 h	14	2, 3, 4, 5, 6, 7, 8, 10, 11, 13, 17, 18^a^, 18^b^, 21	829	0.89	0.49	1.29	4.34	<0.001
	≥ 1 h	8	1, 9, 12, 14, 15, 16, 19, 20	523	1.08	0.22	1.93	2.47	0.013
Outcome measurement time	Immediately	20	2, 4, 5, 6, 7, 8, 9, 10, 11, 12, 13, 14, 15, 16, 17, 18^a^, 18^b^, 19, 20, 21	1,292	0.93	0.50	1.37	4.19	<0.001
	Delayed	2	1, 3	60	0.43	−0.11	0.96	1.56	0.119
Measurement method	Self-report	10	2, 5, 7, 10, 12, 14, 15, 19, 20, 21	686	0.50	0.16	0.85	2.84	0.005
	Observation	12	1, 3, 4, 6, 8, 9, 11, 13, 16, 17, 18^a^, 18^b^	666	1.41	0.66	2.15	3.69	<0.001
Pre-briefing	No	3	6, 9, 12	229	0.08	−0.42	0.58	0.30	0.764
	Yes	19	1, 2, 3, 4, 5, 7, 8, 10, 11, 13, 14, 15, 16, 17, 18^a^, 18^b^, 19, 20, 21	1,123	1.02	0.57	1.48	4.39	<0.001
Debriefing	No	9	3, 5, 6, 8, 9, 12, 16, 17, 19	563	1.39	0.42	2.35	2.81	0.005
	Yes	13	1, 2, 4, 7, 10, 11, 13, 14, 15, 18^a^, 18^b^, 20, 21	789	0.80	0.50	1.09	5.29	<0.001
Other activities	No	19	1, 2, 3, 4, 5, 6, 7, 8, 9, 10, 11, 13, 15, 16, 17, 18^a^, 18^b^, 20, 21	1,094	1.03	0.55	1.50	4.25	<0.001
	Yes	3	12, 14, 19	258	0.15	−0.15	0.44	0.98	0.326
Quality score	< Mean	8	3, 6, 10, 12, 16, 19, 20, 21	480	1.58	0.63	2.54	3.25	0.001
	≥ Mean	14	1, 2, 4, 5, 7, 8, 9, 11, 13, 14, 15, 17, 18^a^, 18^b^	872	0.68	0.26	1.11	3.19	0.001

K, number of analysis sets; N, number of participants; ES, effect size; CI, confidence interval; IRB, institutional review board; Quasi-E, quasi-experimental study; RCT, randomized controlled trial; AR/VR, augmented reality/virtual reality.

Univariate meta-regression identified factors influencing the overall effect. Publication year after 2022 (*Z* = 2.68, *p* = 0.007); having an IRB (*Z* = 5.17, *p* < 0.001); having an fund (*Z* = −2.61, *p* = 0.009); RCT (*Z* = 2.02, *p* = 0.044); intervention duration over than 4 weeks (*Z* = −3.33, *p* < 0.001); intervention session over than 4 sessions (*Z* = −3.01, *p* < 0.001); intervention time/session over than 1 h (*Z* = −5.20, *p* < 0.001); observational measurement rather than self-reporting (*Z* = 3.21, *p* = 0.001); having a pre-briefing before the simulation (*Z* = 3.76, *p* < 0.001); having a debriefing after the simulation (*Z* = −4.41, *p* < 0.001); and having other activities besides the simulation (*Z* = −4.41, *p* < 0.001) had statistically significant effects on nursing competency (Table 5).

**Table 5:** Meta-regression analysis to evaluate competency.

Covariates (Ref.)	Estimate	SE	95% CI		*Z*	*p*-value
			Lower limit	Upper limit		
Year (Ref.: 2018 ~ 2021)	0.10	0.04	0.03	0.18	2.68	0.007
Country (Ref.: Beyond USA)	−0.09	0.19	−0.46	0.28	−0.48	0.631
Language (Ref.: Korean)	−0.12	0.12	−0.35	0.11	−1.03	0.303
School (Ref.: 1)	−0.16	0.18	−0.52	0.19	−0.89	0.376
IRB (Ref.: No)	0.76	0.15	0.47	1.05	5.17	< 0.001
Fund (Ref.: No)	−0.32	0.12	−0.56	−0.08	−2.61	0.009
Research design (Ref.: Quasi-E)	0.25	0.12	0.01	0. 50	2.02	0.044
Participants (Ref.: < 60)	0.01	0.13	−0.25	0.27	0.07	0.945
Intervention type (Ref.: Metaverse)	−0.04	0.14	−0.32	0.23	−0.31	0.756
Facilitator (Ref.: Researcher)	−0.14	0.12	−0.37	0.09	−1.21	0.225
Intervention duration (Ref.: Not reported or <4 weeks)	−0.48	0.14	−0.76	−0.20	−3.33	< 0.001
Intervention session (Ref.: Not reported or <4sessions)	−0.51	0.17	−0.84	−0.18	−3.01	< 0.001
Intervention time/session (Ref.: Not reported or <1 h)	−0.62	0.12	−0.85	−0.39	−5.20	< 0.001
Outcome measurement time (Ref.: Immediately)	−0.21	0.28	−0.75	0.34	−0.74	0.460
Measurement method (Ref.: Self-report)	0.37	0.12	0.15	0.60	3.21	0.001
Pre-briefing (Ref.: No)	0.76	0.15	0.47	1.05	5.17	< 0.001
Debriefing (Ref.: No)	0.45	0.12	0.21	0.68	3.76	< 0.001
Other activities (Ref.: No)	−0.62	0.14	−0.90	−0.35	−4.41	< 0.001
Quality score (Ref.: < Mean)	0.02	0.12	−0.23	0.26	0.14	0.890

Ref, reference; SE, standard error; CI, confidence interval; IRB: institutional review board; Quasi-E, quasi-experimental study.

The exclusion-sensitivity test (38), excluding one study at a time, showed Hedge’s g ranging from 0.67 to 0.94, indicating a moderate to large effect size. The 95% CI (0.36 ~ 0.53, 0.98 ~ 1.36) did not include 0, signifying statistical significance. The effect sizes from the exclusion-sensitivity test were not significantly different from Hedge’s *g* = 0.88, which included all 22 studies (Table 6).

**Table 6:** Exclusion-sensitivity test of the virtual-reality simulation-based intervention.

Study ID	*K*	ES	95% CI		*Z*	*p*-value
			Lower limit	Upper limit		
1	21	0.91	0.48	1.33	4.18	<0.001
2	21	0.89	0.46	1.32	4.05	<0.001
3	21	0.91	0.49	1.32	4.25	<0.001
4	21	0.90	0.47	1.33	4.11	<0.001
5	21	0.94	0.53	1.36	4.47	<0.001
6	21	0.89	0.47	1.32	4.13	<0.001
7	21	0.89	0.46	1.32	4.08	<0.001
8	21	0.76	0.38	1.15	3.88	<0.001
9	21	0.94	0.52	1.36	4.40	<0.001
10	21	0.89	0.46	1.31	4.09	<0.001
11	21	0.89	0.47	1.32	4.11	<0.001
12	21	0.94	0.51	1.36	4.31	<0.001
13	21	0.93	0.50	1.35	4.27	<0.001
14	21	0.92	0.50	1.35	4.23	<0.001
15	21	0.92	0.50	1.34	4.26	<0.001
16	21	0.67	0.36	0.98	4.27	<0.001
17	21	0.91	0.48	1.33	4.18	<0.001
18	21	0.81	0.41	1.22	3.94	<0.001
19	21	0.87	0.45	1.30	4.01	<0.001
20	21	0.91	0.48	1.34	4.16	<0.001
21	21	0.88	0.46	1.30	4.07	<0.001
22	21	0.86	0.44	1.28	4.00	<0.001

K, number of analysis sets; ES, effect size; CI, confidence interval.

### 3.3 Effect of intervention program on secondary outcomes

The secondary outcomes in this study included knowledge, self-efficacy, problem-solving, confidence, and satisfaction. Among these, knowledge, self-efficacy, confidence, and satisfaction exhibited statistically significant changes. After VRS, knowledge and self-efficacy showed significant increases, with moderate effect sizes of Hedge’s *g* = 0.60 (95% CI: 0.07, 1.14) and Hedge’s *g* = 0.53 (95% CI: 0.09, 0.97), respectively. Confidence and satisfaction exhibited substantial increases, with large effect sizes of Hedge’s g = 1.02 (95% CI: 0.48, 1.57) and Hedge’s *g* = 1.35 (95% CI: 0.43, 2.28), respectively (Table 7).

**Table 7:** Effects of virtual reality simulation-based intervention on other variables.

Variables	*K*	Study ID	*N*	ES	95% CI		*Z*	*p*-value
					Lower limit	Upper limit		
Knowledge	6	1, 2, 5, 6, 7, 13	374	0.60	0.07	1.14	2.22	0.027
Self-efficacy	7	2, 8, 9, 12, 14,19, 20	533	0.53	0.09	0.97	2.34	0.019
Problem-solving	3	6, 10, 11	135	0.99	0.00	1.98	1.95	0.051
Confidence	13	1, 2, 5, 7, 9, 10, 11, 15, 16, 17, 18^a^, 18^b^, 21	768	1.02	0.48	1.57	3.66	<0.001
Satisfaction	7	6, 8, 9, 14, 16, 17, 19	433	1.35	0.43	2.28	2.86	0.004

K, number of analysis sets; N, number of participants; ES, effect size; CI, confidence interval.

### 3.4 Publication bias

**Figure 3:**
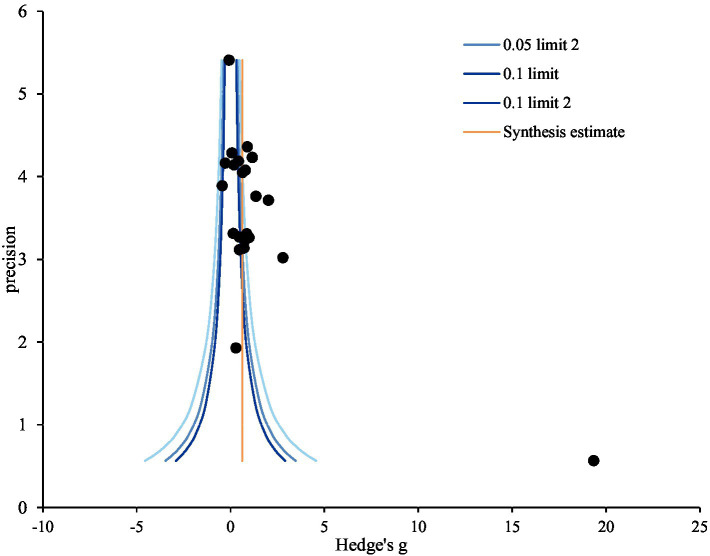
Funnel plot of virtual reality simulation-based intervention on nursing competency. Precision = 1/standard error; 0.05; limit line = 95% confidence limit.

**Figure 4:**
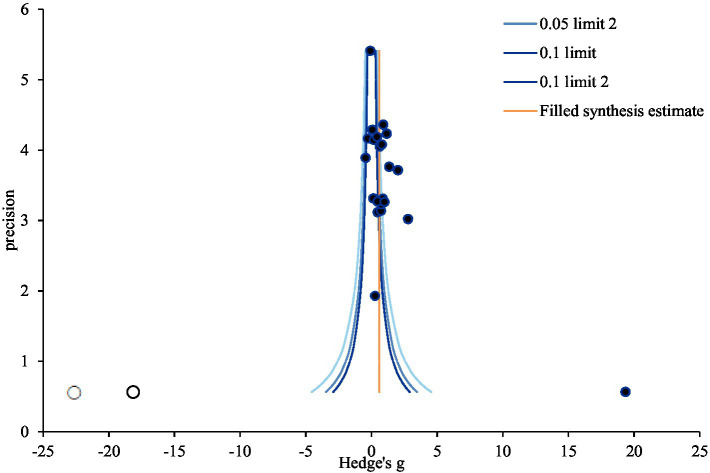
Trim and fill plot of virtual reality simulation-based intervention on nursing competency. Precision = 1/standard error; 0.05; limit line = 95% confidence limit.

To evaluate publication bias, funnel-plot and trim-and-fill plot analyses were conducted. Represented by the black circle, the individual effect sizes of the 22 studies included in the study were asymmetrical— slightly skewed to the right—indicating some potential publication bias (Figure 3). The trim-and-fill plot suggested the addition of one study, represented by the white circle, skewing to the left (Figure 4). Further analysis, The coefficient of the bias was 8.58, indicating the initial value (intercept) and the *p*-value was 0.001. Thus, the null hypothesis was rejected, and the existence of a publication bias could be confirmed. Unlike Egger’s regression test result, Begg’s test for rank correlation (Tau *b* = 0.27, ties = 0; *Z* = 1.75, *p* = 0.080) confirmed the absence of publication bias. Moreover, the trim-and-fill method suggested adding one article; the effect size of the 23 corrected articles was 0.60 (95% CI: 0.49, 0.72). Although the effect size of nursing competency was somewhat smaller after correction than before, it remained statistically significant. In conclusion, this study was deemed free of publication bias (Table 8).

**Table 8:** Publication bias test of virtual reality simulation-based intervention on competency.

Begg’s test	Tau *b*	*K*	S (P-Q)	Ties	*Z*	*p*-value
Standard	0.27	22	63	0	1.78	0.076
Corrected	0.27	22	63	0	1.75	0.080
Egger’s regression test	Coefficient	SE	95% CI		*Z*	*P*-value
			Lower limit	Upper limit		
Intercept	8.58	2.30	4.08	13.08	3.74	0.001
Slope	−1.63	0.62	−2.85	−0.41	−2.62	0.009
Trim and fill method	*K*	ES	95% CI		*Z*	*P*-value
			Lower limit	Upper limit		
Original	22	0.88	0.47	1.29	4.23	<0.001
Corrected	23	0.60	0.49	0.72	10.28	<0.001

Begg’s test for rank correlation; Egger’s regression test for zero intercepts; SE, standard error; CI, confidence interval; K, number of analysis sets; ES, effect size.

## 4 Discussion

In this study, the impact of simulation-based programs on nursing competency demonstrated a significant effect size of 0.88. It’s notable that this simulation-based program yielded encouraging results by positively enhancing nursing competency. This is consistent with similar improvements observed in self-efficacy, a factor linked to nursing competency (19), enhanced knowledge, educational satisfaction, and academic achievement through VR in a hospital environment (39); and improved nursing-process performance (40), heightened critical thinking, clinical performance, and practice satisfaction through vSim for Nursing (41). Additionally, these results partially correlate with those in a study indicating that hands-on training utilizing scenario-based admission management in VR increased learning immersion, learner confidence, and learning satisfaction (7).

In the meta-regression analysis evaluating nursing competency, several factors emerged as influential. First, in cases where the publication year was 2022 or later, nursing competency was found to be significantly improved compared to studies that received IRB approval, compared to studies published before then. In the evolving landscape of clinical practice, recent emphasis on patient safety and rights has shifted the focus toward observing nursing behavior rather than direct patient care (42). This shift underscores the active implementation of simulation-based education, suggesting a more systematic adaptation of teaching methods and educational systems to enhance nursing competency compared to previous approaches. Moreover, studies with an intervention duration not reported or one of less than 4 weeks demonstrated a significant effect on nursing competency compared to those lasting more than 4 weeks. In cases of intervention with fewer than four sessions, competency was significantly improved compared to intervention sessions with four or more sessions. Similarly, interventions with time per session not reported or those lasting less than 1 h were associated with a significant improvement in nursing competency compared to those lasting more than 1 h. These findings suggest that shorter, more intensive interventions may be more effective in enhancing nursing competency through VRS. Establishing short-term intensive courses could thus be a meaningful approach. Even in the case of pre-briefings, which are recognized for their utility, the introduction and assignment of roles and expectations during pre-briefings may not be optimal. This is because simulation anxiety is linked to higher levels of extraneous cognitive load (43). Further investigation into the timing and temporal aspects of these activities is warranted to optimize their effectiveness. Therefore, further research specifically focusing on the temporal aspect is deemed necessary to comprehensively understand its implications.

Furthermore, pre-briefing before simulation emerged as a significant factor contributing to the improvement of nursing competency compared to that in the control group. This is consistent with the recognized importance of pre-briefing in face-to-face simulations, in which it influences simulation readiness (44). Given that most included studies conducted virtual pre-briefing activities individually, such as pre-briefing lessons and quizzes, the findings imply that virtual pre-briefing can be actively utilized with comparable effectiveness in face-to-face simulations. Various pre-briefing methods, including role rubrics, are currently under development (45). Further research will be necessary to ascertain the effectiveness of these diverse pre-briefing approaches.

Moreover, this study identified that post-simulation debriefing had a more significant effect of improving nursing competency compared to non-simulation debriefing. This could be attributed to the characteristic of VRS that enables repeated and reflective learning through debriefing with immediate feedback, thus providing learner-customized learning (46). The ability to facilitate individual improvement in nursing competency through immediate feedback is consistent with previous studies emphasizing the effectiveness and importance of debriefing in simulation (47). While debriefing in a virtual setting may differ from team interaction, reflection, and discussion in a face-to-face simulation, the results underscore the crucial role of debriefing in VRS situations.

Competency improved significantly when observation was measured rather than self-report. Role assignment in nursing simulation often elicits significant anxiety stemming from uncertainty, performing in front of faculty and peers, and social evaluation (45). Moreover, many individuals perceive themselves as lacking proficiency, particularly in terms of nursing competency. Consequently, self-reported improvements in nursing competency may underestimate actual progress observed through objective evaluation. Hence, effective communication and encouragement regarding the significance of simulation are vital when implementing simulation programs.

Nursing competency was statistically significantly improved when compared to those who did not engage in any other activities other than simulation. Other activities take as much time, which suggests that core simulation activities are important for improving nursing competency. Non-simulation activities, denoting the absence of activities other than simulation, exhibited a significant effect on nursing competency. While non-simulation activities may improve competencies such as team cooperation, communication, or empathy, they were not associated with improvements in nursing competency. This suggests that clear simulation content, along with pre-briefing and debriefing activities tailored to enhance nursing competency, directly influence this competency.

Meanwhile, several variables did not demonstrate a statistically significant effect of improving nursing competency. The country, number of centers, funding status, research design, and all the variables related to the operation of the intervention program (participants, intervention type, facilitator, intervention session, and outcome-measurement time), as well as the quality score, did not show significant differences in improving nursing competency. The inconsistency in trends observed across these variables can be attributed to the diverse definitions and measurements of nursing competency utilized in the included studies. This variability in research outcomes underscores the absence of a standardized measurement tool for nursing competency, potentially leading to increased heterogeneity in results.

Furthermore, the secondary outcomes measured alongside nursing competency in this study included knowledge, self-efficacy, problem-solving, confidence, and satisfaction. Among these, knowledge and confidence demonstrated statistically significant improvement. These variables, particularly knowledge and confidence, are closely related to nursing competency and can concurrently improve with it in VRS. Conversely, self-efficacy, problem-solving, and satisfaction did not show significant improvement. This is consistent with previous research indicating that VR nursing education improves knowledge (48) and increases learning satisfaction (49) but does not enhance technical skills (48) or significantly impact self-efficacy (49). This suggests that while VRS is effective in improving knowledge-related competencies, consistent improvements in self-efficacy, problem-solving, and satisfaction may depend on its design and utilization.

Given that learning immersion through simulation has been demonstrated to impact the development of clinical-nursing competence (50), and VR-based programs have been effective in improving cognitive performance, such as theoretical knowledge, through realism (51), VRS holds promise in nursing education. However, the results of this study underscore the need to carefully consider elements that are more challenging to implement in virtual situations than in face-to-face scenarios. Therefore, further research, such as systematic reviews and meta-analyses exploring other variables in VRS, is recommended for a more comprehensive understanding of its impact on nursing education.

VR-based nursing education represents an innovative field that has not been previously explored. These simulators offer a range of environments that transcend physical constraints, enabling participants to immerse themselves within the virtual space (52). It’s crucial for educators responsible for program development to grasp the distinctions between virtual reality and reality to facilitate effective education.

This study underscores the significance of pre-briefing and debriefing elements in VR-based simulation, highlighting the importance of their organization. Rather than focusing solely on operational time or the duration of the simulation itself, the key lies in how these elements are implemented for optimal educational outcomes. Additionally, when assessing effectiveness, we advocate for a combined approach utilizing both self-reported evaluations and objective evaluations through observation or assessment.

### 4.1 Limitations of the study

This study acknowledges several limitations. First, there is variability in reporting randomization methods among the included studies, with some providing comprehensive discussions on the topic while others lack detailed information on the methods employed. Second, the diverse interpretations and definitions of nursing competency across the included studies may introduce variability in the study outcomes. Third, the absence of a standardized measurement tool for nursing competency could contribute to increased heterogeneity. Fourth, the selection criteria for the studies analyzed, which included only those published in English or Korean and reported precise means, standard deviations, and sample sizes, could lead to selection bias and limit the generalization of our study results. Additionally, the studies encompass sample sizes from different countries, further contributing to overall heterogeneity. To enhance the robustness of future research and validate the effectiveness of interventions for nursing students, larger sample sizes and higher-quality studies are recommended.

## 5 Conclusion

The meta-analysis of nursing competency in VRS revealed the latter’s effectiveness in enhancing nursing competency. Notably, the incorporation of key elements from face-to-face simulation, such as pre-briefing and debriefing, significantly improved nursing competency compared to scenarios in which these elements were absent. This study suggests the importance of reflecting core simulation elements in virtual simulations and underscores the need to enhance the quality of pre-briefing and debriefing in virtual contexts. Moreover, the findings suggest that intensively operating VRS over a short period could be more effective in improving nursing competency. This implies the significance of considering the effectiveness of short-term intensive courses for nursing-competency improvement within virtual spaces. The study findings provide valuable insights for the design of VRS aimed at enhancing nursing competency.

## Data availability statement

The original contributions presented in the study are included in the article/supplementary material, further inquiries can be directed to the corresponding authors.

## Author contributions

M-KC: Conceptualization, Data curation, Formal analysis, Methodology, Visualization, Writing – original draft, Writing – review & editing. MK: Conceptualization, Data curation, Funding acquisition, Project administration, Resources, Supervision, Validation, Writing – original draft, Writing – review & editing.

## References

[1] Han D-L . Nursing students’ perception of virtual reality (VR) and needs assessment for virtual reality simulation in mental health nursing. J Digit Contents Soc. (2020) 21:1481–7. doi: 10.9728/dcs.2020.21.8.1481

[2] Foronda CL Fernandez-Burgos M Nadeau C Kelley CN Henry MN. Virtual simulation in nursing education: a systematic review spanning 1996–2018. Simul Healthc. (2020) 15:46–54. doi: 10.1097/SIH.0000000000000411 32028447

[3] Foronda CL . What is virtual simulation? Clin Simul Nurs. (2021) 52:8. doi: 10.1016/j.ecns.2020.12.004, PMID: 38642632

[4] Shin H Rim D Kim H Park S Shon S. Educational characteristics of virtual simulation in nursing: an integrative review. Clin Simul Nurs. (2019) 37:18–28. doi: 10.1016/j.ecns.2019.08.002, PMID: 34769972

[5] Foronda C Gattamorta K Snowden K Bauman EB. Use of virtual clinical simulation to improve communication skills of baccalaureate nursing students: a pilot study. Nurse Educ Today. (2014) 34:e53–7. doi: 10.1016/j.nedt.2013.10.007, PMID: 24231637

[6] Irwin P Coutts R. A systematic review of the experience of using second life in the education of undergraduate nurses. J Nurs Educ. (2015) 54:572–7. doi: 10.3928/01484834-20150916-05, PMID: 26431517

[7] Kim YJ . Development and application of scenario-based Admission Management VR contents for nursing students. J Korea Soc Comput Inf. (2021) 26:209–16. doi: 10.9708/jksci.2021.26.01.209

[8] Butt AL Kardong-Edgren SK Ellertson A. Using game-based virtual reality with haptics for skill acquisition. Clin Simul Nurs. (2018) 16:25–32. doi: 10.1016/j.ecns.2017.09.010

[9] Jung A Kwon E Seo J. Effects of nursing skills simulation program using virtual reality (VR) on learning flow, nursing skills confidence, nursing skills performance and usability verification. J Korea Acad-Ind Coop Soc. (2022) 23:127–35. doi: 10.5762/KAIS.2022.23.11.127

[10] Kim JW . Virtual reality (VR) based sustainable food education contents for elementary school students. Korean Assoc Pract Arts Edu. (2019) 32:45–63. doi: 10.24062/kpae.2019.32.4.45

[11] Ahn MK . The development and effects of metaverse-based core nursing skill contents of vital signs measurements and subcutaneous injections for nursing students. J Korean Acad Soc Nurs Educ. (2022) 28:378–88. doi: 10.5977/jkasne.2022.28.4.378

[12] Lee JS . Implementation and evaluation of a virtual reality simulation intravenous injection training system. Int J Environ Res Public Health. (2022) 19:5439. doi: 10.3390/ijerph19095439, PMID: 35564835 PMC9105754

[13] Ahn MK Lee CM. Development and effects of head-mounted display-based home-visits virtual reality simulation program for nursing students. Korean Soc Nurs Sci. (2021) 51:465–77. doi: 10.4040/jkan.21051 34497255

[14] Rossler KL Sankaranarayanan G Duvall A. Acquisition of fire safety knowledge and skills with virtual reality simulation. Nurse Educ. (2019) 44:88–92. doi: 10.1097/NNE.0000000000000551, PMID: 29847356 PMC6252293

[15] Aebersold M Voepel-Lewis T Cherara L Weber M Khouri C Levine MD . Interactive anatomy, augmented virtual simulation training. Clin Simul Nurs. (2018) 15:34–41. doi: 10.1016/j.ecns.2017.09.008, PMID: 29861797 PMC5978424

[16] An J Oh J Park K. Self-regulated learning strategies for nursing students: a pilot randomized controlled trial. Int J Environ Res Public Health. (2022) 19:9058. doi: 10.3390/ijerph19159058, PMID: 35897439 PMC9331953

[17] Chang CY Panjaburee P Chang SC. Effects of integrating maternity VR based situated learning into professional training on students’ learning performances. Interact Learn Environ. (2022) 2022:1–15. doi: 10.1080/10494820.2022.2141263

[18] Kim MS Jeong HC. The effects and adaptation of augmented reality–based intradermal injection practice education for nursing students. J Korean Soc Simul Nurs. (2022) 10:93–104. doi: 10.17333/JKSSN.2022.10.2.93

[19] Ha YO Kwon SJ Kim J Song JH. Effects of nursing skills practice using VR (virtual reality) on competency and confidence in nursing skills, learning self-efficacy, and satisfaction of nursing students. J Ind Converg. (2022) 20:47–55. doi: 10.22678/JIC.2022.20.4.047

[20] You H Yang B. The effects of virtual reality simulation scenario application on clinical competency, problem solving ability and nursing performance confidence. J Korea Acad Ind Coop Soc. (2021) 22:116–26. doi: 10.5762/KAIS.2021.22.9.116

[21] Bae YS Shin KM. Effects of virtual reality simulation of core fundamental nursing skills for intravenous fluid infusion on nursing students. Korean J Care Manag. (2023) 46:95–119. doi: 10.22589/kaocm.2023.46.95

[22] Song YM . Online and blended learning application in psychiatric and mental health nursing practice program among nursing students. J Learn Cent Curric Instr. (2021) 21:289–303. doi: 10.22251/jlcci.2021.21.11.289

[23] Raman S Labrague LJ Arulappan J Natarajan J Amirtharaj A Jacob D. Traditional clinical training combined with high fidelity simulation based activities improves clinical competency and knowledge among nursing students on a maternity nursing course. Nurs Forum. (2019) 54:434–40. doi: 10.1111/nuf.12351, PMID: 31093991

[24] Cho IY Yun JY Moon SH. Development and effectiveness of a metaverse reality-based family-centered handoff education program in nursing students. J Pediatr Nurs. (2024) 76:176–91. doi: 10.1016/j.pedn.2024.02.005, PMID: 38412709

[25] Lee E Baek G. Development and effects of a virtual reality simulation nursing education program combined with clinical practice based on an information processing model. Comput Inform Nurs. (2023) 41:1016–25. doi: 10.1097/CIN.0000000000001051, PMID: 37647622

[26] Kim J Heo N. Effect of augmented reality smart glasses-based nursing skills training for nursing students’ medication administration safety competency: a quasi-experimental study. J Korean Acad Fundam Nurs. (2023) 30:449–58. doi: 10.7739/jkafn.2023.30.4.449

[27] Park S Yoon HG. Effect of virtual-reality simulation of indwelling catheterization on nursing students’ skills, confidence, and satisfaction. Clin Simul Nurs. (2023) 80:46–54. doi: 10.1016/j.ecns.2023.05.001

[28] Karaduman GS Basak T. Is virtual patient simulation superior to human patient simulation: a randomized controlled study. CIN Comput Inform Nu. (2023) 41:467–76. doi: 10.1097/CIN.0000000000000957 36633879

[29] Moon SH . Metaverse based emergency nursing educational program using V-story. Crisis. (2023) 19:79–89.

[30] Lee JJ . The effect of virtual reality simulation training on critical thinking disposition, clinical competency, and self-efficacy of nursing students. J Korea Acad Ind Coop Soc. (2023) 24:390–7. doi: 10.5762/KAIS.2023.24.12.390

[31] Ahn MK . Development and effects of metaverse-based CPR training. J Digit Contents Soc. (2023) 24:1347–52. doi: 10.9728/dcs.2023.24.6.1347

[32] Tufanaru C Munn Z Aromataris E Campbell J Hopp L. Chapter 3. Systematic reviews of effectiveness In: Aromataris E Munn Z, editors. JBI manual for evidence synthesis (2020). JBI; 2024.

[33] Borenstein M Hedges LV Higgins JPT Rothstein HR. Introduction to Meta-analysis. West Sussex, UK: John Wiley & Sons (2009).

[34] Higgins JPT Thompson SG. Quantifying heterogeneity in a meta-analysis. Statist Med. (2002) 21:1539–58. doi: 10.1002/sim.1186 12111919

[35] Higgins JPT , Green SE (2011). Available at: http://www.cochrane-handbook.org (Accessed September 13, 2023).

[36] Mavridis D Salanti G. How to assess publication bias: funnel plot, trim-and-fill method and selection models. Evid Based Ment Health. (2014) 17:30. doi: 10.1136/eb-2013-101699, PMID: 24477535 PMC13404685

[37] Brydges CR . Effect size guidelines, sample size calculations, and statistical power in gerontology. Innov. Aging. (2019) 3:igz036. doi: 10.1093/geroni/igz036, PMID: 31528719 PMC6736231

[38] Bown MJ Sutton AJ. Quality control in systematic reviews and meta-analyses. Eur J Vasc Endovasc Surg. (2010) 40:669–77. doi: 10.1016/j.ejvs.2010.07.011, PMID: 20732826

[39] Kim MG Kim HW. The effects of classes using virtual reality simulations of the hospital environment on knowledge of the hospital environment, academic self-efficacy, learning flow, educational satisfaction and academic achievement in nursing students. J Korean Acad Fundam Nurs. (2021) 28:520–9. doi: 10.7739/jkafn.2021.28.4.520

[40] Lim JH . The effect of virtual reality simulation education on nursing process competency. J Digit Converg. (2021) 19:401–9. doi: 10.14400/JDC.2021.19.9.401

[41] Kim S Kim MJ. Effect of learner-centered virtual reality simulation education. J Digit Converg. (2022) 20:705–13. doi: 10.14400/JDC.2022.20.4.705

[42] Yang SJ Chae MJ. Effect of nursing students’ practical training on nursing simulation for blood transfusion recipients using online virtual reality. J Digit Contents Soc. (2024) 25:143–51. doi: 10.9728/dcs.2024.25.1.143

[43] Fredericks S ElSayed M Hammad M Abumiddian O Istwani L Rabeea A . Anxiety is associated with extraneous cognitive load during teaching using high-fidelity clinical simulation. Medical education. Online. (2021) 26:1994691. doi: 10.1080/10872981.2021.1994691, PMID: 34710001 PMC8555543

[44] Brennan BA . The impact of self-efficacy based prebriefing on nursing student clinical competency and self-efficacy in simulation: an experimental study. Nurse Educ Today. (2022) 109:105260. doi: 10.1016/j.nedt.2021.105260, PMID: 34973554

[45] Dodson TM Reed JM. Enhancing simulation preparation: Presimulation role rubrics and expert Modeling videos. Clin Simul Nurs. (2024) 87:101498. doi: 10.1016/j.ecns.2023.101498

[46] Lim S Yeom YR. The effect of education integrating virtual reality simulation training and outside school clinical practice for nursing students. J Converg Inf Technol. (2020) 10:100–8.

[47] Loomis A Dreifuerst KT Bradley CS. Acquire, apply, and retain knowledge through debriefing for meaningful learning. Clin Simul Nurs. (2022) 68:28–33. doi: 10.1016/j.ecns.2022.04.002

[48] Chen FQ Leng YF Ge JF Wang DW Li C Chen B . Effectiveness of virtual reality in nursing education: a meta-analysis. J Med Internet Res. (2020) 22:e18290. doi: 10.2196/18290, PMID: 32930664 PMC7525398

[49] Padilha JM Machado PP Ribeiro A Ramos J Costa P. Clinical virtual simulation in nursing education: randomized controlled trial. J Med Internet Res. (2019) 21:e11529. doi: 10.2196/11529, PMID: 30882355 PMC6447149

[50] Kim HW Suh EY. Nursing students’ immersion experience in a comprehensive simulation scenario using high-fidelity human patient simulator among nursing students: a phenomenological study. J Mil Nurs Res. (2012) 30:89–99.

[51] Shorey S Ng ED. Use of virtual reality simulation among nursing students and registered nurses: a systematic review. Nurse Educ Today. (2021) 98:104662. doi: 10.1016/j.nedt.2020.104662, PMID: 33203545

[52] Hwang YJ Jeong JY Jeong YM. A study on the feasibility of introducing XR in nursing education Core fundamental nursing skills. J Digit Contents Soc. (2023) 24:775–83. doi: 10.9728/dcs.2023.24.4.775

